# Repositioning liothyronine for cancer immunotherapy by blocking the interaction of immune checkpoint TIGIT/PVR

**DOI:** 10.1186/s12964-020-00638-2

**Published:** 2020-09-07

**Authors:** Xiuman Zhou, Jiangfeng Du, Hongfei Wang, Chunxia Chen, Ling Jiao, Xiangrui Cheng, Xiaowen Zhou, Shaomeng Chen, Shanshan Gou, Wenshan Zhao, Wenjie Zhai, Junhui Chen, Yanfeng Gao

**Affiliations:** 1 Intervention and Cell Therapy Center, Peking University Shenzhen Hospital, Shenzhen Peking University-The Hong Kong University of Science and Technology Medical Center, Shenzhen, 518035 China; 2 School of Life Sciences, Zhengzhou University, Zhengzhou, 450001 China; 3 School of Pharmaceutical Sciences (Shenzhen), Sun Yat-sen University, Shenzhen, 518107 China

**Keywords:** PVR, TIGIT, Small molecule compound, Virtual screening, Liothyronine, Cancer immunotherapy

## Abstract

**Background:**

Inhibitors targeting immune checkpoint were proved effective in cancer immunotherapy, such as PD-1/PD-L1 blockade. The novel immune checkpoint TIGIT/PVR plays critical roles in suppressing the anti-tumor effects of CD8^+^ T and NK cells, and dual blockade of TIGIT/PVR and PD-1/PD-L1 by antibody can elicit synergistic effects in tumor models and clinical trials. However, small molecules for TIGIT/PVR blockade have not been investigated.

**Methods:**

The expression of PVR in tumors were analyzed by using TCGA, Oncomine and GEO database, and in cancer cell lines examined by flow cytometry. Natural product compounds were docked to PVR for virtual screening by using the software Molecular Operating Environment (MOE). Candidate compounds were further tested by biolayer interferometry-based binding assay, microscale thermophoresis assay and cell based blocking assay. The in vitro activity of the candidate compound was determined by MTT, peripheral blood mononuclear cells (PBMCs) activation assay and coculture assay. The anti-tumor effects and mechanism were also investigated by using MC38 tumor-bearing mice model and immune cell depletion tumor model.

**Results:**

PVR was over-expressed in many tumor tissues and cancer cell lines, making it a promising therapeutic target. Through virtual screening, binding, and blocking assay, liothyronine was discovered to bind PVR and block the interaction of TIGIT/PVR. Liothyronine could enhance the function of CD4^+^ and CD8^+^ T cells in PBMCs. Besides, in the Jurkat-hTIGIT and CHOK1-hPVR coculture assay, liothyronine could reverse the IL-2 secretion inhibition resulted by TIGIT/PVR ligation. Although had no influence on the proliferation of tumor cells in vitro, liothyronine could significantly inhibit tumor growth when administrated in vivo, by enhancing CD8^+^ T cell infiltration and immune responses in the tumor bearing mice. The immune cell depletion model showed that the anti-tumor effects of liothyronine depends on CD4^+^ T cells, CD8^+^ T cells and NK cells.

**Conclusions:**

A small molecule liothyronine was discovered to serve as a potential candidate for cancer immunotherapy by blocking the immune checkpoint TIGIT/PVR.

**Video abstract**

**Graphical abstract:**

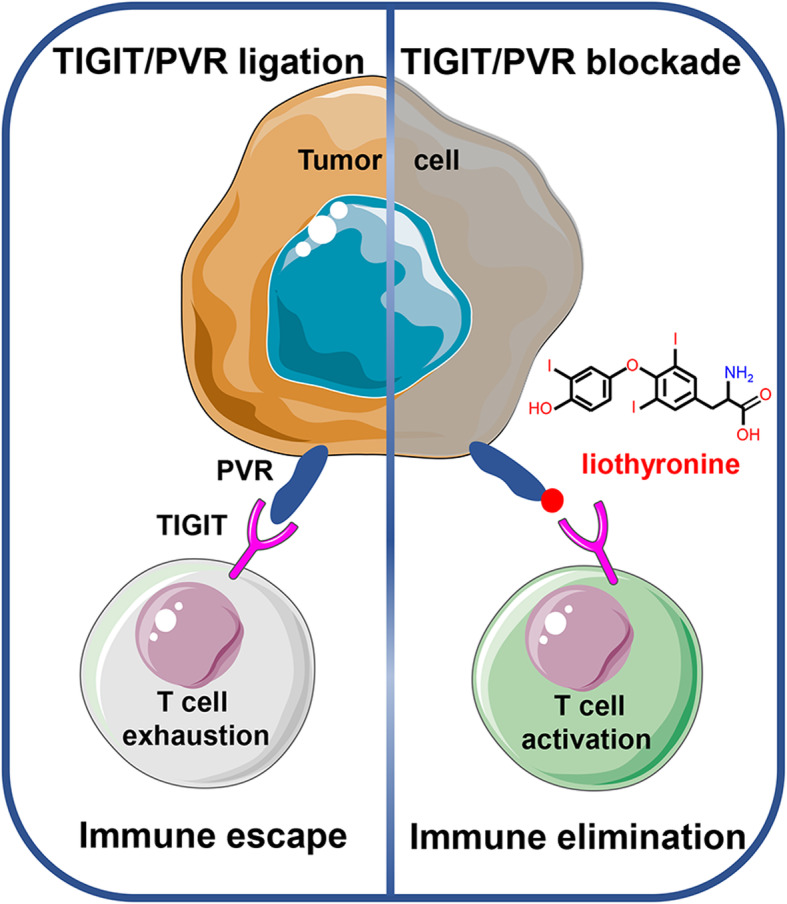

## Background

Immune checkpoint blockade based cancer immunotherapy has achieved unprecedented success, represented by PD-1/PD-L1 blockade [1]. However, the response rate of PD-1/PD-L1 blockade therapy varies greatly among patients with different types of tumors, which is urgent to be improved [2]. Another treatment dilemma is that adaptive resistance is observed in patients who initially exhibit effective response to PD-1/PD-L1 antibody [3]. Therefore, various combination strategies were investigated to get better therapeutic efficacy, such as PD-1/PD-L1 blockade combined with radiotherapy or chemotherapy [4]. On the other hand, great progress has achieved in discovering novel immune checkpoints which could also synergize with PD-1/PD-L1 and were non-redundant in restricting the anti-tumor response of immune cells, such as TIGIT, LAG-3, TIM-3 and CD47 [5]. Among these, TIGIT was found to be expressed not only on immune cells (such as CD8^+^ T and NK cells), but also on tumor cells, which making it a potential target for cancer immunotherapy [6].

The dominant ligand of TIGIT, poliovirus receptor PVR (also known as CD155 and Nectin like-5), was firstly identified for its ability to serve as the cellular receptor for poliovirus [7]. PVR is a cell surface glycoprotein with V-C2-C2 domain, and belongs to both immunoglobulin superfamily and Nectin/Necl family. Initial studies mainly focused on its role in mediating virus invasion and as an adhesion-related molecule for tumor invasion and migration [8, 9]. Subsequently, PVR was identified as the ligand of co-stimulatory molecule CD226 and co-inhibitory immune checkpoint TIGIT, indicating more attention should be paid to its importance in cancer immunity [10, 11]. It has been reported that PVR was highly expressed in a variety of tumors, including colorectal cancer, breast cancer, small cell lung cancer, head and neck squamous cell carcinoma, melanoma and gastric cancer [12–15]. PVR interacts with the immunoglobulin variable (IgV) domain of TIGIT and forms a tetramer to deliver inhibitory signals [16]. TIGIT/PVR ligation could disrupt the granule polarization and cytotoxicity of NK cells and suppress the anti-virus and anti-tumor activity of CD8^+^ T cells [17, 18]. TIGIT blockade alone or with PD-1/PD-L1 or TIM-3 could significantly restore the function of CD8^+^ T cells and inhibit tumor growth in both tumor models and clinical trials [2, 18–20]. Besides, PVR blockade could reduce the experimental metastasis of osteosarcoma [21].

The development of agents for immune checkpoint blockade mainly focused on antibodies. In recent years, an increasing number of low molecular weight inhibitors such as peptides and small molecules have been investigated and showed great application prospects. They have shown strong tissue penetration, weak immunogenicity, and can be easily modified. The first proteolytic resistant D-enantiomer peptide targeting PD-L1, and a cyclic peptide of LAG-3 developed by our group exhibited significant tumor inhibition as candidates for cancer immunotherapy [22, 23]. Besides, small molecules targeting immune checkpoints are under rapid development with great progress [24, 25]. Orally bioavailable small molecule CA170 for dual blockade of PD-L1 and VISTA is undergoing clinical trials [26]. However, small molecules for TIGIT/PVR blockade have not been investigated.

The resolution of the crystal structure of TIGIT/PVR complex makes docking based virtual screening targeting PVR feasible, which could reduce the amount of small molecule compounds tested by biochemical assays, and significantly improve the discovery efficiency. Natural products from plants, animals, marine life, bacteria, and other organisms are important resources for modern drug discovery and have played significant role in anticancer drug discovery and development [27]. The biological relevance and structural diversity make natural products good starting points for drug discovery.

In this study, we explored the expression of PVR analyzed by bioinformatics tools of TIMER, Oncomine, GEO database, and flow cytometry. By docking based virtual screening, we screened the natural product compounds targeting PVR. The compounds were further screened by biolayer interferometry-based binding assay and cell based blocking assay. The anti-tumor activity and potential mechanism of the candidate compound liothyronine were explored.

## Methods

### Gene expression analysis

TIMER is a comprehensive resource for systematic analysis of immune infiltrates across diverse cancer types (https://cistrome.shinyapps.io/timer/) [28]. It has seven modules for analysis of 32 cancer types from The Cancer Genome Atlas (TCGA) including gene, survival, mutation, somatic CNA, differential gene expression, correlation and estimation. Here, the expression level of PVR was analyzed between various types of tumor and normal tissues with the differential gene expression module. The gene expression level was displayed with log_2_RSEM.

The expression level of PVR between tumor and normal tissues was also identified using the Oncomine database (https://www.oncomine.org/resource/login.html) [29]. Relevant statistical indicators were set as follows: *P* < 1 × 10^− 4^, fold change > 2 and gene ranking in the top 10%. Besides, the outlier analysis of colorectal cancer, esophagus cancers, gastric cancers, renal cancers and leukemia were performed.

Gene expression data of PVR in colorectal primary tumors, normal tissues or polyps were downloaded from the National Institutes of Health Gene Expression Omnibus (GEO) database (https://www.ncbi.nlm.nih.gov/geo/). Datasets GSE37182 (probe ID: ILMN_1677305 for PVR), GSE10972 (probe ID: ILMN_1677305 for PVR) and GSE41258 (probe ID: 212662_at for PVR) were analyzed.

### Cell culture

Murine colorectal cancer cell lines CT26 and MC38 were cultured in Dulbecco’s Modified Eagle Medium (DMEM). Human colorectal cancer cell lines HT29 and SW620, Chinese hamster ovary cell lines CHO-K1, CHO-K1 transfected with TIGIT (referred to as CHOK1-hTIGIT or CHOK1-mTIGIT), CHO-K1 transfected with PVR (referred to as CHOK1-hPVR) and Jurkat transfected with TIGIT (referred to as Jurkat-hTIGIT) were cultured in Roswell Park Memorial Institute (RPMI) 1640 medium. Human peripheral blood mononuclear cells (PBMCs) isolated from healthy donors were cultured in Iscove’s Modified Dulbecco’s Medium (IMDM). All the cells were cultured at 37 °C with 5% CO_2_ under fully humidified conditions with above media (GIBCO, Grand Island, USA) supplemented with 10% fetal bovine serum FBS (BI, USA), 100 U/mL penicillin (Solarbio, China) and 100 μg/mL streptomycin (Solarbio, China).

### Flow cytometry

Harvested cells (3 × 10^5^) were washed with pre-cold FACS buffer (pH 7.2 PBS containing 2% FBS), and incubated with indicated antibodies in a final volume of 50 μL FACS buffer for 30 min. Cells were then washed with FACS buffer and re-suspended with 200 μL FACS buffer for flow cytometry detection. Antibodies anti-human PVR PE (clone: 2H7CD155), mouse IgG1 κ isotype control (clone: P3.6.2.8.1) were used for the detection of PVR on CHOK1-hPVR and human colorectal cell lines HT29 and SW620. Anti-mouse PVR APC (clone: TX56) and rat IgG2a κ Isotype Control (clone: eBR2a) were used for the detection of PVR on murine colorectal cell lines CT26 and MC38. Anti-human TIGIT PE (clone: MBSA43), mouse IgG1 κ isotype control (clone: P3.6.2.8.1), anti-mouse TIGIT PE (clone: GIGD7) and rat IgG2a κ isotype control (clone: eBR2a) were used for the verification of the over-expression of human or mouse TIGIT on CHOK1 and Jurkat cells. All the antibodies were purchased from eBioscience. The data were acquired by a BD FACSCalibur with CELLQuest™ software and analyzed by FlowJo.

### Virtual screening

The crystal structure of the human TIGIT/PVR complex has been elucidated through X-ray crystallography with the resolution of 2.9 Å (PDB ID:3UDW) and deposited in the Protein Data Bank database (PDB) (https://www.rcsb.org/). The 3D structure of PVR (chain C of 3UDW) was selected for docking followed by structure optimization with the software Molecular Operating Environment (MOE) (version: 2016.08) (Chemical Computing Group ULC, Canada). By optimization, the hydrogen number was corrected, the system was charged, two residues of the C-terminal were deleted and Protonate 3D was performed for protonation. The binding area was determined by selecting the residues in the distance of 4.5 Å. Binding pocket was selected by the established in silico methods by the Site Finder module of MOE. An in-house natural products library with 638 compounds were used for virtual screening. Prior to docking to the prepared structure of PVR, the structures of these compounds were washed to remove extraneous salts or adjust protonation states, in order to ensure that each structure is in a form suitable for subsequent docking. Database minimization was also performed. Molecular docking was conducted with the algorithm of MOE. The top scoring ligands were judged by scores and inspected manually with specific attention to their conformations and the interactions with the pocket. Twenty-two compounds were selected for in vitro analysis and purchased from commercial suppliers (TargetMol).

### Biolayer interferometry-based (BLI) binding assay

BLI experiments were performed in PBS supplemented with 0.1% (w/v) BSA, 0.002% (v/v) Tween 20 and 1% DMSO, with an Octet RED96 instrument (FortéBio), operating at 25 °C. The biotinylated PVR carrying a C-terminal AVITAG (CD5-H82E3, ACRO Biosystem) was loaded to saturation onto Super Streptavidin (SSA) biosensors (Pall/FortéBio), and blocked with 200 μL biotin (5 μg/mL) for 60 s. Before the assay, the PVR-labeled SSA sensors were pre-soaked in binding buffer for 30 min. The twenty-two small molecule compounds were preliminary screened for binding to PVR at a single concentration of 100 μM. The association of PVR with the eight small molecule compounds with response over than 0.03 nm was then measured at a series of concentrations. The dissociation was monitored by washing the ligand biosensors from the analyte solution to binding buffer for 60 s. Double reference-subtracted sensorgrams were analyzed using Data Analysis 9.0 (Pall/Forte Bio), and binding responses at the end of the association phase were reported.

### Microscale thermophoresis (MST) binding assay

MST assay was performed using the Monolith NT.115 system to determine the affinity of SMC 14 to human and mouse PVR protein. Human PVR (CD5-H5223, ACRO Biosystem) and mouse PVR (50259-M08H, Sino Biological) were both fused with a His tag. The protein was labeled with Red-NHS647 dye (NanoTemper Technologies GmhH, Germany) and used for the subsequent test. SMC 14 was 2-fold serially diluted from 100 μM and 16 samples were subsequently tested. Equal volume of Red-NHS647 dye-labeled protein was incubated with the SMC 14 for 5 min at room temperature, the mixture was loaded onto standard capillaries for detection. The K_D_ values were calculated with analysis software (MO.Affinity Analysis v2.2.4).

### Cell based blocking assay

CHOK1-TIGIT cells stably expressing human or mouse TIGIT were established by lenti-viral infection followed by puromycin selection according to the previous report [6]. Cell based blocking assay was performed according to the previous report [30]. Briefly, PVR-Fc protein was incubated with a series dilution of small molecule compounds or control buffer on ice for 30 min, human PVR-Fc (CD5-H82F6, ACRO Biosystem) and mouse PVR-Fc (50259-M03H, Sino Biological) were used at concentrations of 10 and 40 ng/test, respectively. Then the mixture of PVR protein and compounds were incubated with CHOK1-TIGIT cells for another 30 min, followed by incubation with detection antibody anti-human IgG-PE (eBioscience) for 30 min. Cells were then washed with FACS buffer to remove the unbound fluorescent antibodies, acquired on a FACS Caliber™ (BD Biosciences) and analyzed by FlowJo. To test the specificity of candidate compounds, similar blocking assays were performed by using PD-1 (PD-L1-Fc, 10,084-H02H, Sino Biological) and CD47 (Sirpα-Fc, SIA-H52A8, ACRO Biosystem) over-expressed CHOK1 cells. Cells incubated with corresponding Fc tagged protein and anti-human IgG-PE served as positive control, while cells only incubated with anti-human Fc-PE served as negative control. The mean fluorescent intensity (MFI) was recorded for calculating the blocking efficiency as the formula:

$$ Blocking\%=\frac{\mathrm{MFI}\ \mathrm{of}\ \mathrm{positive}\ \mathrm{control}-\mathrm{MFI}\ \mathrm{of}\ \mathrm{tested}\ \mathrm{compounds}}{\mathrm{MFI}\ \mathrm{of}\ \mathrm{positive}\ \mathrm{control}}\times 100\%. $$

### Cell viability assay

The cytotoxic effects of indicated compound on MC38 cells were determined by MTT assay. MC38 cells were seeded into 96-well plates at a density of 3000 cells/well and allowed to grow for adhesion. Cells were treated with medium with 1% DMSO as negative control or with a series dilution of indicated compound at a concentration range of 12.5 to 100 μM. Cell viability was ascertained using MTT reagent (Sigma) dissolved in 5 mg/mL PBS and incubated at 37 °C for 4 h in the following 3 days. Formazan crystals formed by viable cells were dissolved in 150 μL DMSO in each well. MTT reduction was quantified by measuring the absorbance at 490 nm.

### PBMC activation assay

To determine whether the small compounds could stimulate lymphocyte activation, Peripheral blood mononuclear cells (PBMCs) activation assay was performed as previously reported [31, 32]. Briefly, the PBMCs were isolated from blood samples of healthy donors by the Ficoll-Paque density gradient centrifugation, and re-suspended in an IMDM medium containing 10% FBS. PBMCs were seeded into round bottom 48-well plates at a density of 4 × 10^5^ cells/well, and stimulated with stimulatory antibodies of 1 μg/mL human anti-CD3 (clone: OKT3, eBioscience) and 0.5 μg/mL human anti-CD28 (clone: CD28.2, eBioscience). Indicated compound (100 μM) or medium with 1% DMSO as negative control were tested for the ability to enhance the activation of lymphocytes in PBMCs. Three days later, an intracellular cytokine staining assay was performed. Cells were incubated with protein transport inhibitor (Containing Brefeldin A) (BD Biosciences) for 4 h, and stained with antibodies of human anti-CD4 PerCP-Cy5.5 (clone: OKT4, eBioscience) and human anti-CD8 APC (clone: SK1, eBioscience) on ice for 30 min. Then, cells were fixed and permeabilized each for 30 min with the Foxp3/Transcription Factor Staining Buffer Set (eBioscience) according to the manufacture. Permeabilized cells were then stained with human anti-IFN-γ PE antibody (clone: 4S.B3, eBioscience) or an isotype control.

### Coculture assay

Jurkat-hTIGIT and CHOK1-hPVR cells were cocultured to determine the ability of SMC 14 for TIGIT/PVR blockade. Briefly, CHOK1-hPVR were seeded into 24-well plates at a density of 1 × 10^5^ cells/well and allowed for cell adhesion. Jurkat-hTIGIT were added into the plates pre-cultured with CHOK1-hPVR at a density of 2 × 10^5^ cells/well with the effector: target ratio of 2:1. Jurkat-hTIGIT cells were stimulated with the anti-human CD3 (1 μg/mL) and anti-human CD28 (0.5 μg/mL). SMC 14 (100 μM) were added in the coculture system, and a functional anti-TIGIT antibody (clone: A15153A, Biolegend) served as the positive control. After 44 h of coculture, the protein transport inhibitor was added and incubated for another 4 h. Then the cells were harvested, washed, and followed by fixation and permeabilization. Afterwards, the permeabilized Jurkat-hTIGIT cells were stained with anti-human IL-2 APC (MQ1-17H12, Biolegend) or isotype control antibody for 30 min at 4 °C.

### Anti-tumor experiment and ex vivo assays

All mice experimental procedures were approved by the Ethics Committee of Zhengzhou University. Six-week-old female C57BL/6 mice (Vital River Laboratories, China) were maintained in a specific pathogen-free facility. Food and water were supplied ad libitum. Mice were acclimatized for about 1 week before the experiment. C57BL/6 mice were subcutaneously (s.c.) injected on the right flank with 1 × 10^6^ syngeneic MC38 cells in 200 μL PBS. One week later, tumor-bearing mice were randomized into groups of negative control (normal saline with 3% DMSO and 0.5% Tween-80), 0.5, 1.5, 5 and 15 mg/kg of compounds or anti-TIGIT and isotype control groups. Mice were treated with 200 μL control or indicated compound through intraperitoneal (i.p.) injection every other day for 2 weeks, or with 200 μg anti-TIGIT (clone:1G9, Bioxcell) or isotype control mouse IgG (I5381, Sigma) through i.p. injection every 3 days for 2 weeks. Tumor sizes were measured using a digital caliper, and tumor volumes were calculated as the formula:

$$ V=\frac{1}{2}\times \mathrm{a}\ \left(\mathrm{length}\right)\times \mathrm{b}\ \left(\mathrm{width}\right)\times \mathrm{c}\ \left(\mathrm{height}\right) $$

For indicated immune cell depletion models, MC38 mice were injected with 250 μg anti-CD4 (clone: GK1.5), 200 μg anti-CD8 (clone: YTS169.4) or matched rat IgG control (I4131, Sigma), 200 μg anti-NK1.1 (clone: PK136) or matched mouse IgG control (I5381, Sigma) the day before the start of tumor inoculation, as well as weekly thereafter, resulting in a total of 3 injections per mouse. The depletion efficacy was confirmed by flow cytometry analysis with the blood sample of mice treated with indicated antibodies for 6 days. Mouse anti-CD45 FITC (clone: 30-F11, eBioscience), anti CD3 PerCP-eFluor710 (clone: 17A2, eBioscience), anti-CD8α APC (clone: 53–6.7, eBioscience), anti-CD4 APC (clone: GK1.5, eBioscience) and anti-NK1.1 PE (clone: PK136, eBioscience) were used.

Upon completion of treatment, MC38 tumor-bearing mice were sacrificed, and the tumors were dissected and digested by 100 U/mL collagenase IV (Invitrogen) and 100 U/mL Dnase I (Sigma) for 60 min, and then filtered through a 70 μm nylon cell strainer to single cell suspension. The cells were incubated with mouse anti-CD45 FITC (clone: 30-F11, eBioscience), mouse anti-CD3 PerCP-eFluor710 (clone: 17A2, eBioscience), and mouse anti-CD8α APC (clone: 53–6.7, eBioscience) to detect the frequency of infiltrating CD8^+^ T cells. For functional assay, the tumor-infiltrating lymphocytes were isolated from tumor cell suspension by discontinuous Percoll density gradients (40 and 70%) (GE Healthcare). The isolated tumor-infiltrating lymphocytes, splenocytes and draining lymph node cells prepared by gentle mechanical disruption were stimulated with or without 20 ng/mL phorbol 12-myristate 13-acetate (PMA, Sigma) and 1 μMionomycin (Sigma) in the presence of protein transport inhibitor for 4 h. The following procedure of intracellular cytokine staining assay was performed as mentioned above, and the antibodies for the staining were mouse anti-CD3 PerCP-eFluor710, mouse anti-CD8α PE, and mouse anti-IFN-γ APC (clone: XMG1.2, eBioscience).

### Statistical analysis

The data were shown as means ± SEM, and the statistical analysis was conducted with paired or unpaired Student’s *t*-test for the differences between groups. *P < 0.05, ** *P* < 0.01, and *** *P* < 0.001 were considered statistically significant.

## Results

### The expression analysis of PVR in tumor tissues and cell lines

**Fig. 1:**
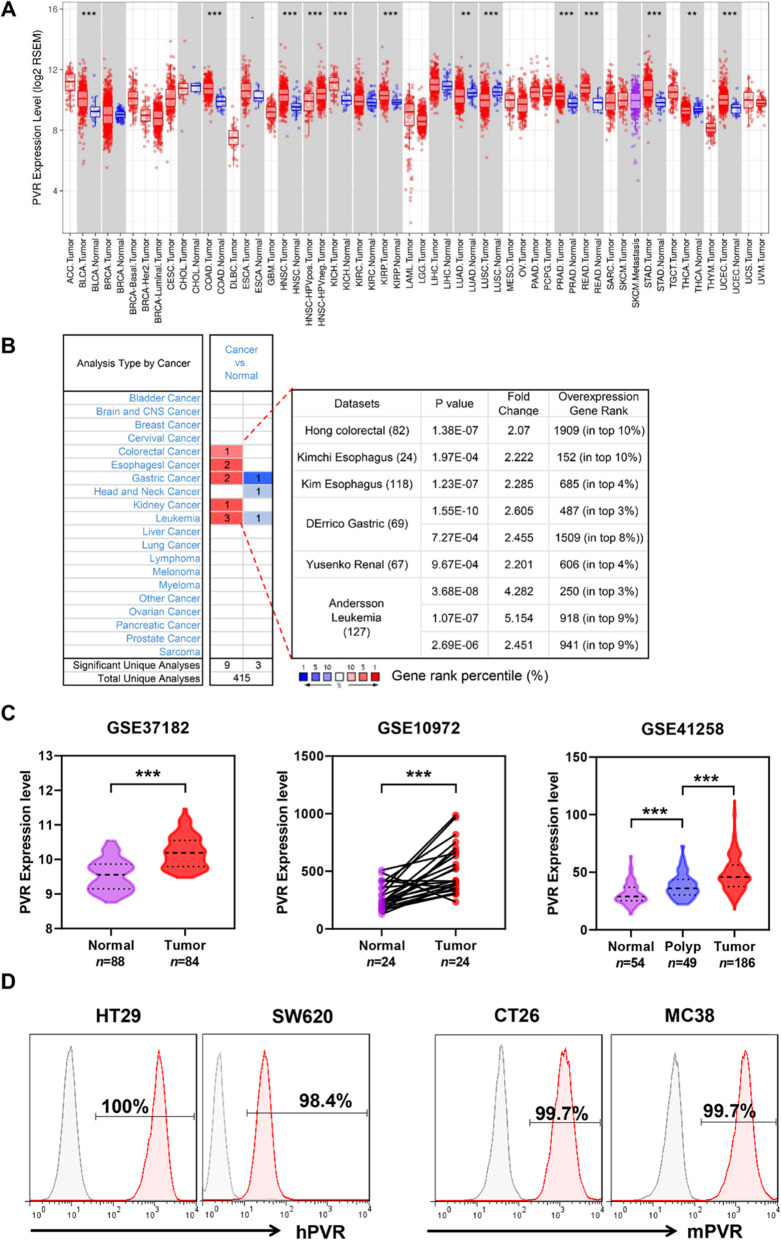
The expression level of PVR in tumor tissues and cell lines. **a** Human PVR expression levels in different tumor types from TCGA database were determined by TIMER (*P < 0.05, **P < 0.01, ***P < 0.001). **b** Increased or decreased PVR expression in datasets of different cancers compared with normal tissues in the Oncomine database. (Threshold: *P* < 0.001, fold change> 2, gene ranking of top 10%). **c** PVR expression level in colorectal cancers, polyps and normal tissues from GEO cohorts. Statistical analysis was conducted with unpaired Student’s *t*-test for datasets GSE37182 and GSE41258, and conducted with paired Student’s *t*-test for dataset GSE10972. ***, *P* < 0.001. **d** Flow cytometry analysis of PVR expression on human and murine colorectal cell lines. The red-shaded histogram represents anti-human PVR PE (left). The red-shaded histogram represents anti-mouse PVR APC (right). The gray-shaded histogram represents the matched isotype control. Representative histogram of three independent experiments were shown

To confirm the expression of PVR in tumors, we firstly analyzed the data from TCGA using the tools TIMER. As shown in Fig. 1a, PVR expressed in head and neck cancer (HNSC), lung adenocarcinoma (LUAD) and stomach cancer (STAD), which was consistent with the previous study [13, 15, 33]. In addition, it also expressed across a broad spectrum of cancers including bladder cancer (BLCA), endometrioid cancer (UCEC), esophageal cancer (ESCA), kidney chromophobe (KICH), Kidney papillary cell carcinoma (KIRP), prostate cancer (PRAD), thyroid cancer (THCA), and especially in colon cancer (COAD) and rectal cancer (READ) within 50 datasets. Meanwhile, PVR expression was also analyzed in Oncomine, which PVR expressed higher in colorectal cancers, esophagus cancers, gastric cancers, renal cancers and leukemia than corresponding normal tissues. The gene rank information was shown in Fig. 1b. The further analysis focused on colorectal cancer observed that PVR expressed higher in colorectal cancer tissues than normal tissues to both non-paired samples of GSE37182 and 24 paired samples of GSE10972. More importantly, by analyzing the GSE41258 dataset, we observed that the expression level of PVR was gradually upregulated during the tumor development from normal colon to polyps and primary tumors (Fig. 1c). Besides, the flow cytometry results also showed that PVR expressed on both human and murine colorectal cancer cell lines (Fig. 1d). These results suggested that PVR might be a potential target for the treatment of colorectal cancer.

### Virtual screening of small molecule compounds targeting PVR

**Fig. 2:**
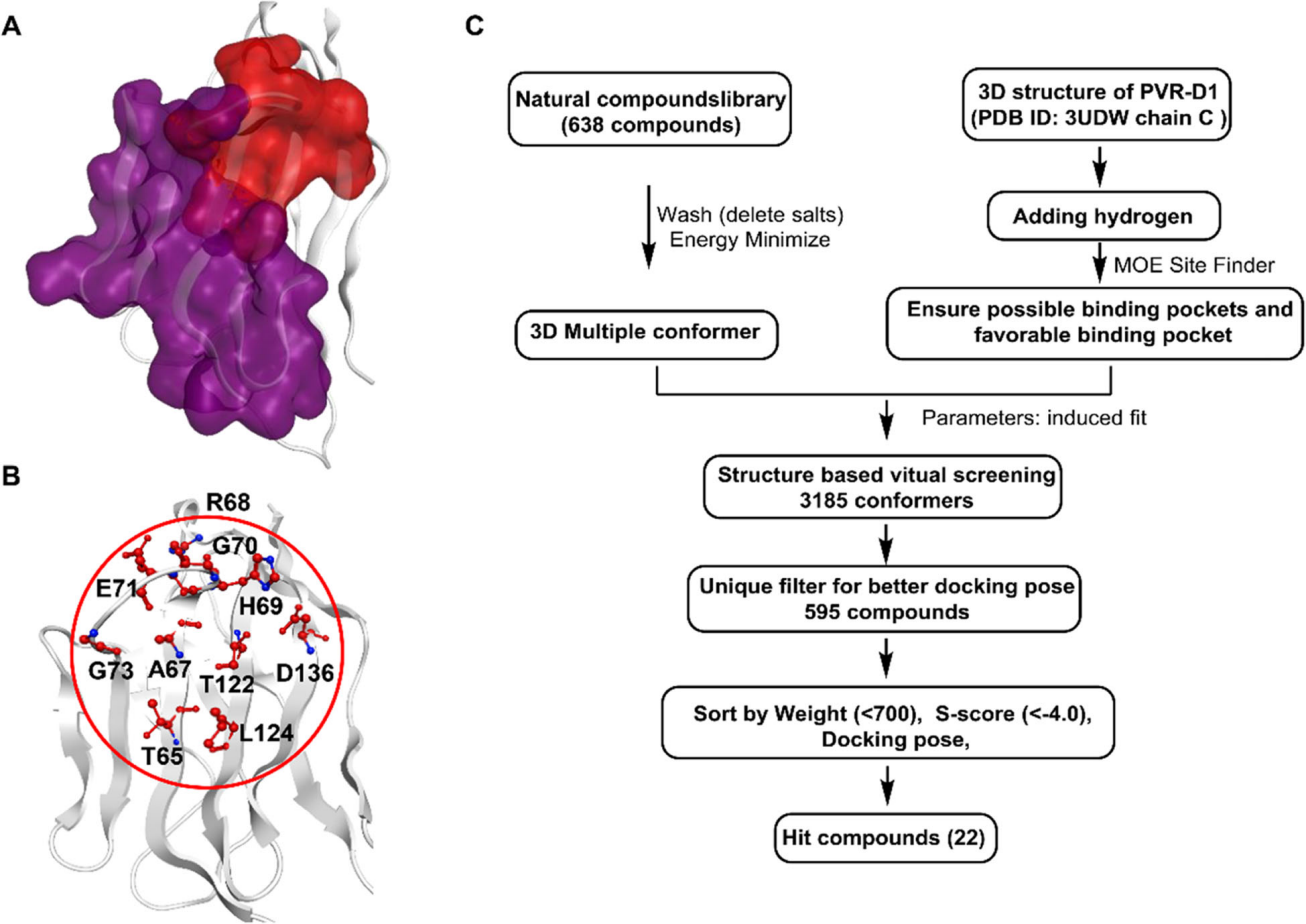
Virtual screening of small molecule compounds from the natural product library targeting PVR. **a** The structure of PVR from PDB database (PDB ID: 3UDW), with the binding area of TIGIT/PVR interaction labeled in purple, and the binding pocket of compounds identified by MOE labeled in red. **b** The detail of interacting residues of the binding pocket. **c** The procedure of screening small molecule compounds targeting PVR

The crystal structure of TIGIT/PVR complex had been released, which provides a structure basis for the drug discovery targeting PVR. To screen small molecule compounds blocking TIGIT/PVR interaction, the structure of PVR was prepared by correcting the hydrogen numbers, deletion of two residues in the C-terminal, and followed by protonation. The residues of PVR in a distance of 4.5 Å to TIGIT (chain A of 3UDW) was labeled as the binding area (Fig. 2a). Five binding pockets were determined using the Site Finder module of MOE (supplemental Table 1). The binding pocket occupies different regions of PVR, and the second pocket is the only one close to the binding area of TIGIT/PVR and has overlaid part with the binding area, upon which we may discover small molecule compounds occupy the area in purple and interfere the interaction of TIGIT and PVR. The residues form this binding pocket were selected for subsequent molecular docking (Fig. 2b). The compounds of natural product library were washed, performed energy minimization, and docked to the structure prepared PVR. The compounds were screened as the procedure shown in Fig. 2c. With comprehensive consideration of the molecular weight, S-score which represents the binding energy, and especially the conformations and interactions with the pocket, twenty-two candidate compounds were selected for further biochemical assays. The detail information of candidate compounds was shown in supplemental Table 1.

### The biochemical evaluation of candidate compounds by BLI

**Fig. 3:**
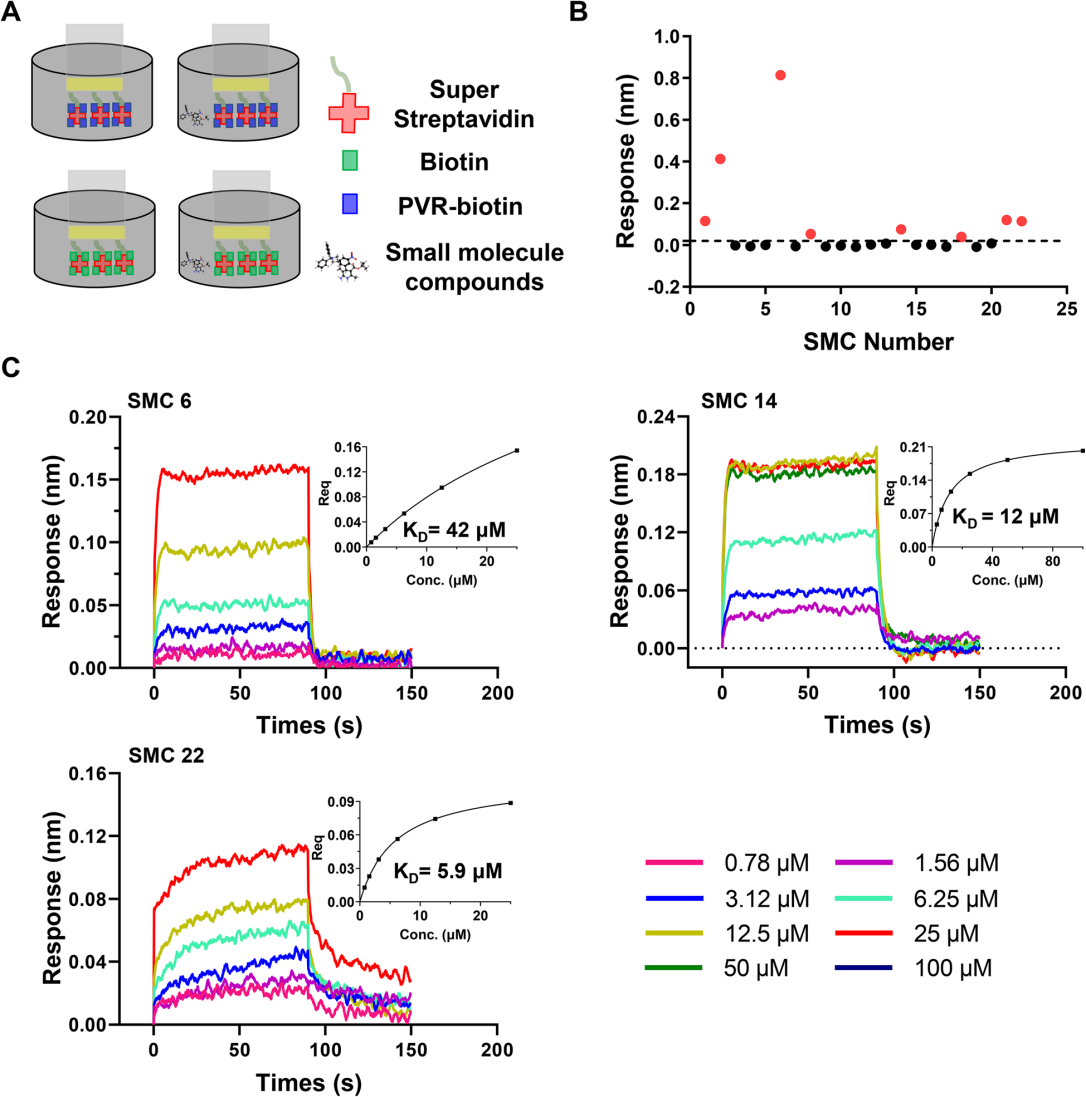
Evaluation of the binding affinity of small molecule compounds towards PVR. **a** Schematic representation of the double reference-subtracted analysis system. **b** The responses of the small molecule compounds preliminarily screened at a single concentration of 100 μM. The compounds with responses over than 0.03 nm were labeled red. **c** The dissociation curves and K_D_ values of the three compounds with high affinity to PVR were shown. Data are representative of three independent experiments

The binding affinity of all the candidate compounds targeting PVR were tested by the double reference-subtracted BLI assay as depicted in Fig. 3a. The test was initialed with a primary screening with a single concentration of 100 μM, and eight compounds exhibited relative high response above 0.03 nm (Fig. 3b). The eight compounds were further tested with a series of concentrations for obtaining the K_D_ values to PVR protein, and three compounds, SMC 6, SMC 14 and SMC 22 possessed high affinities to PVR with K_D_ values of 42 μM, 12 μM, and 5.9 μM, respectively (Fig. 3c). The other five compounds, including SMC 2 with the relatively good response, could not fit a determined K_D_ value with the binding and dissociation curves at series concentrations.

### Screening of the candidate compounds by cell based blocking assay

**Fig. 4:**
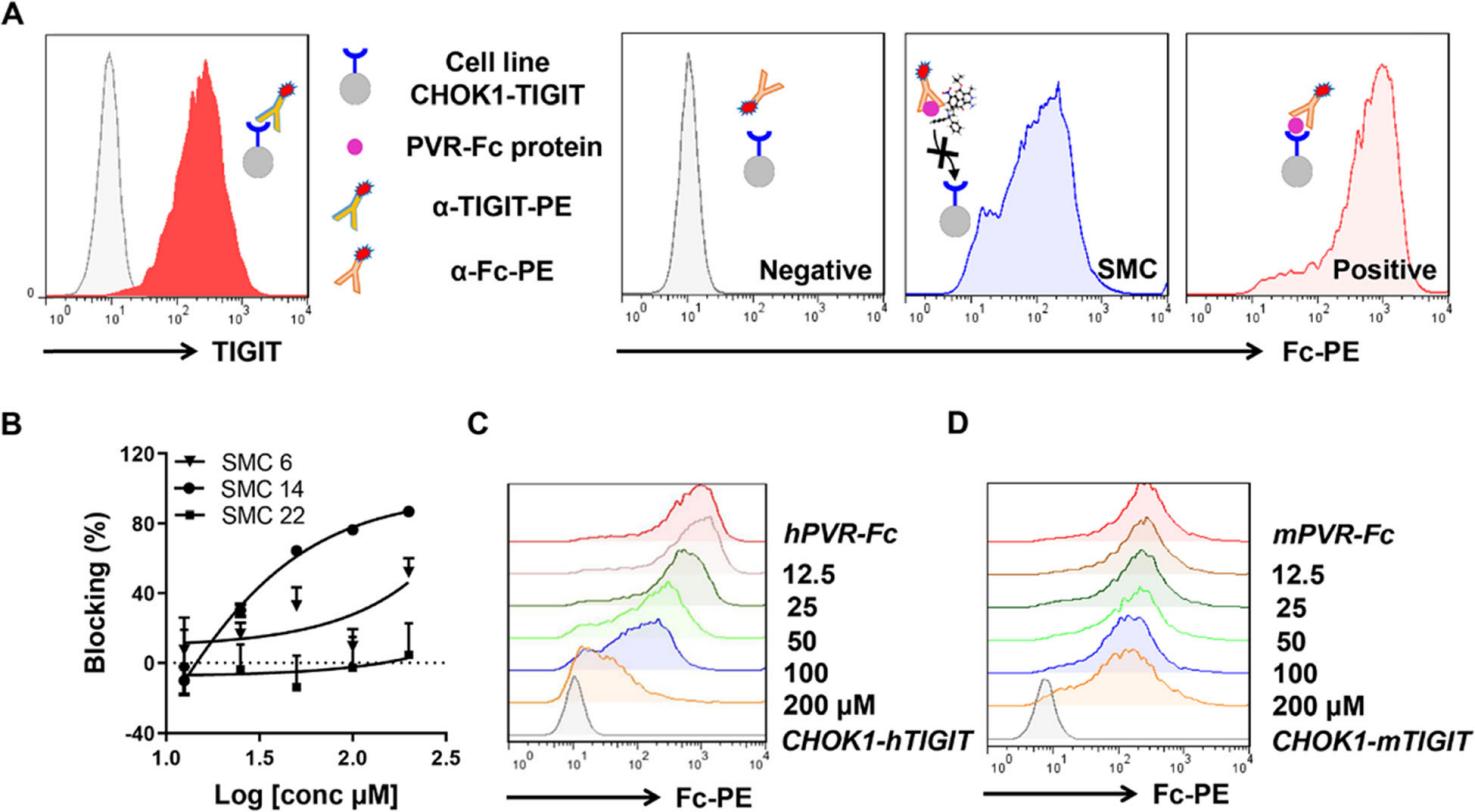
The blocking efficacy of TIGIT/PVR interaction by small molecule compounds. **a** Schematic representation of cell based blocking assay. The TIGIT expression level on CHOK1-hTIGIT cells were confirmed (Left). The representative FACS histogram of tested compounds, cells only incubated with anti-human IgG-PE served as the negative and cells incubated with human PVR-Fc protein served as the positive control (Right). **b** The dose dependent blocking curves of SMC 6, SMC 14 and SMC 22 were shown. (c and d) The representative FACS histogram of blocking assay of SMC 14 with CHOK1-hTIGIT **c** and CHOK1-mTIGIT cells **d** were shown. Data are representative of at least three independent experiments

The ability of these three compounds for blocking TIGIT/PVR were further tested by cell based blocking assay with CHOK1-hTIGIT cells as depicted in Fig. 4a. Human PVR protein with an Fc tag was used to bind to the cell membrane TIGIT and served as the positive control. In the blocking assay, SMC 22 exhibited extremely low blocking efficacy, and SMC 6 had a moderate blocking efficacy of 52% at the highest concentration. SMC14 (liothyronine) could significantly block the ligation of PVR and TIGIT in a dose dependent manner with the IC_50_ of 6.1 μM (Fig. 4b). The blockade of human TIGIT/PVR by liothyronine at different concentrations was shown in Fig. 4c. Further, the blocking efficacy of mouse TIGIT/PVR was tested by using mPVR protein and CHOK1 cells overexpressing mTIGIT, and liothyronine could also block the interaction of mouse PVR and TIGIT (Fig. 4d). Considering that the blocking efficacy of mouse TIGIT/PVR is lower than that of human TIGIT/PVR, the affinity of SMC 14 to human and mouse PVR was also tested by MST assay. SMC 14 could bind to mPVR with the K_D_ value of 2.64 μM and bind to hPVR with a lower K_D_ value of 0.36 μM (Fig. S1), and the difference of the affinity may explain the blocking efficacy of human and mouse TIGIT/PVR interaction. As the binding model shown in Fig. S2, liothyronine could be docked to PVR in the binding pocket and interact with the residues at the binding area of TIGIT. Considering that most of the immune checkpoint molecules interact with their ligands or receptors through the IgV-like domain, and shared certain structural similarities, the activities of liothyronine in PD-1/PD-L1 and CD47/Sirpα cell based blocking system were also tested. Liothyronine could not block the interaction of PD-1/PD-L1 nor CD47/Sirpα, indicating its specificity targeting TIGIT/PVR (Fig. S3a, b).

### The effects of liothyronine on colorectal tumor cells and immune cells in vitro

**Fig. 5:**
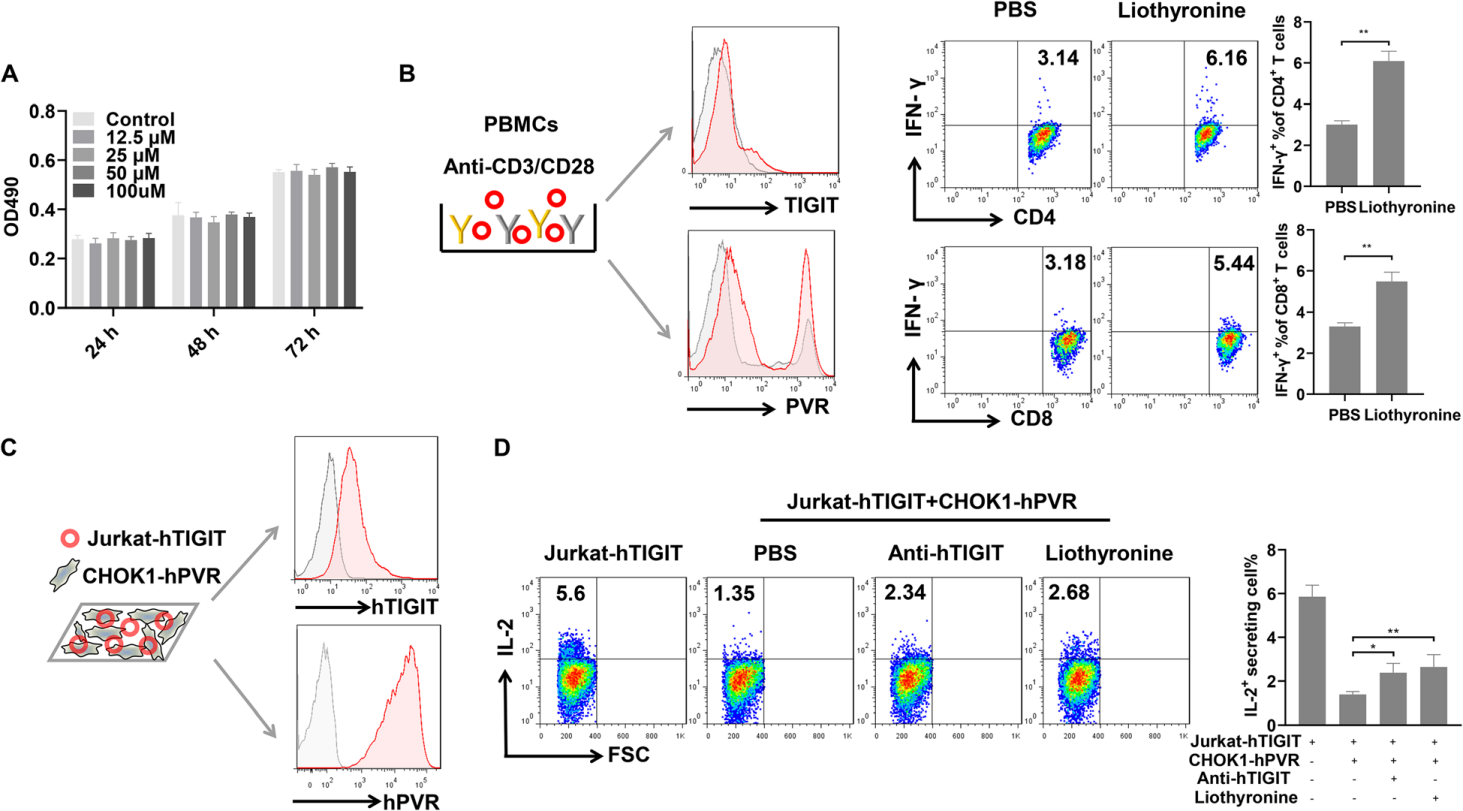
The effects of liothyronine on tumor cells and lymphocytes in vitro. **a** The effects of liothyronine on MC38 tumor cell viability measured by MTT. There were no significant differences between the groups. Data are representative of at least three independent experiments. **b** PBMCs were activated with anti-CD3 and anti-CD28 stimulatory antibodies. The expression of TIGIT and PVR on activated PBMCs were detected by flow cytometry. PBMCs without activated were labeled in gray, activated PBMCs were represented in red. Flow cytometry analysis of intracellular IFN-γ staining of CD4^+^ T (upper) and CD8^+^ T cells (lower) in PBMCs. Representative results of three donors were shown. (**P < 0.01) **c** Schematic diagram of the cocultured assay using Jurkat-hTIGIT and CHOK1-hPVR cells. Representative histograms of TIGIT expression on Jurkat-hTIGIT cells and PVR expression on CHOK1-hPVR cells were shown. **d** Jurkat-hTIGIT cells were stimulated with anti-CD3 and anti-CD28 stimulatory antibodies for 48 h, 1 μg/mL of anti-TIGIT and 100 μM liothyronine was added, the frequencies of IL-2 secreting Jurkat-hTIGIT were detected. Representative flow cytometry plots and summary data were shown. (*P < 0.05, **P < 0.01) Data are representative of at least three independent experiments

Then, the in vitro cytotoxic activity in colorectal tumor cells and immune modulating effects on PBMCs of liothyronine were determined. The results suggested that liothyronine did not impact the cell viability of MC38 cells at the tested concentrations (Fig. 5a). To determine the functional result of TIGIT/PVR blockade by SMC 14, a PBMCs activation assay was performed. Similar as described previously, [11] TIGIT was expressed on the PBMCs stimulated by anti-CD3 and anti-CD28 antibodies (Fig. 5b). Since PVR was also expressed on PBMCs, the TIGIT/PVR ligation would suppress the T cell activation. The addition of liothyronine in the stimulated PBMCs could significantly restore the secretion of IFN-γ by CD4^+^ and CD8^+^ T cells (Fig. 5b). Besides, a coculture assay was also performed using the CHOK1-hPVR cells and TIGIT overexpressing Jurkat cells which was widely used as T cells for immune checkpoint blockade assays (Fig. 5c). TIGIT/PVR interaction could significantly inhibit the IL-2 secretion of Jurkat-hTIGIT cells, while a functional anti-TIGIT antibody and SMC 14 could significantly reverse the inhibition (Fig. 5d). Therefore, our results revealed that liothyronine did not affect the proliferation of tumor cells, but significantly enhanced the function of T cells, indicating liothyronine has the typical characteristics of immune checkpoint blockers.

### Liothyronine could inhibit tumor growth and elicit immune response in mice

**Fig. 6:**
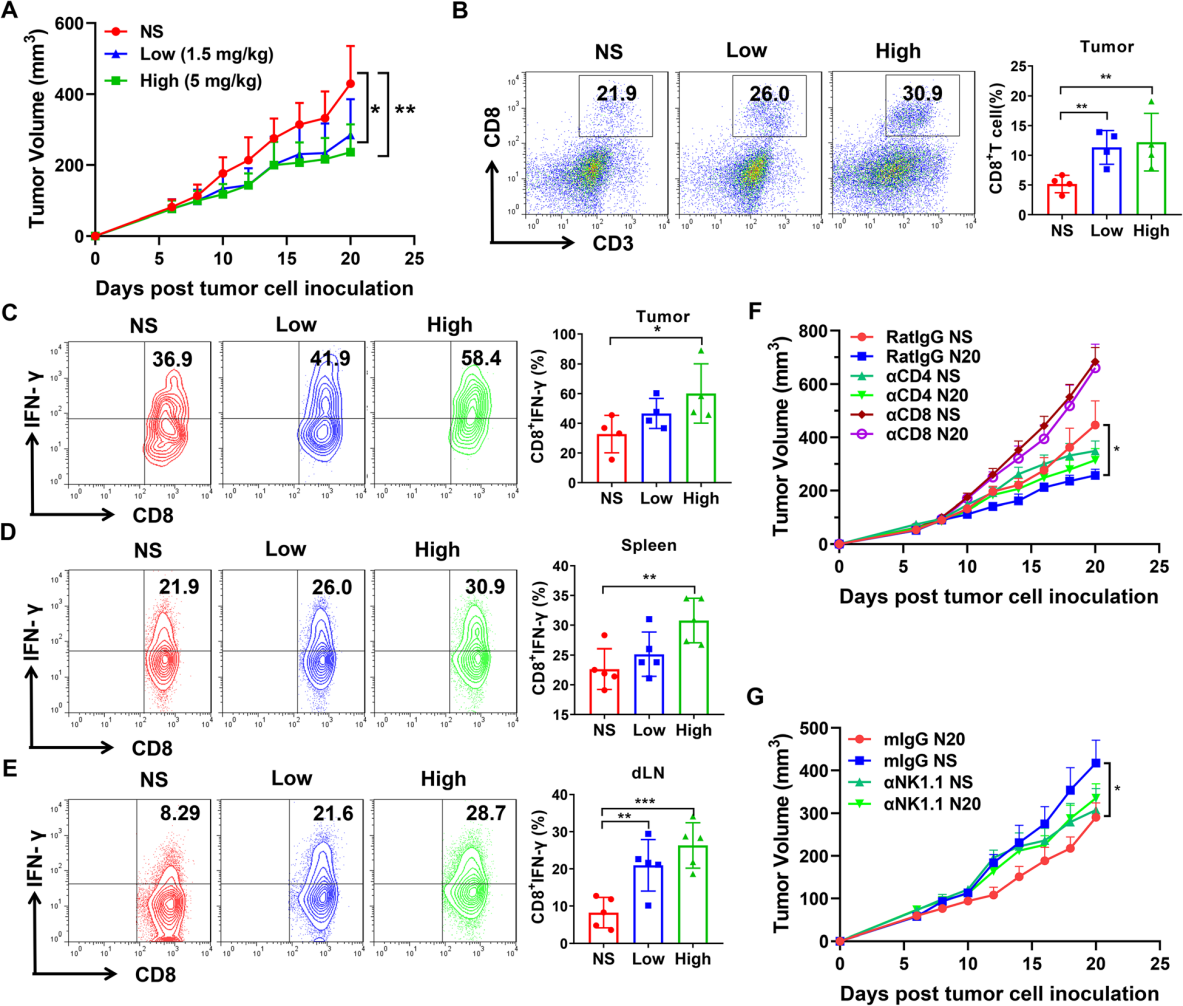
The anti-tumor effects of liothyronine on MC38 tumor model. **a** Tumor growth curve of MC38 tumor-bearing mice treated with normal saline, low dose (1.5 mg/kg) and high dose (5 mg/kg) of liothyronine by intraperitoneal injection every other day. (*n* = 7, *P < 0.05, **P < 0.01). **b** Flow cytometry analysis of the proportion of CD8^+^ T cells infiltration at the tumor site of tumor-bearing mice. Intracellular IFN-γ staining assay of tumor infiltrating lymphocytes **c**, splenocytes **d** and tumor draining lymph node **e**. **f** Tumor growth curves of tumor-bearing mice injected with rat IgG, CD4 or CD8 depletion antibodies treated by control or liothyronine (5 mg/kg). (*n* = 4, *P < 0.05). **g** Tumor growth curves of tumor-bearing mice injected with mouse IgG or NK1.1 depletion antibody treated by control or liothyronine (5 mg/kg). (*n* = 4, *P < 0.05)

To explore the anti-tumor effects of liothyronine in mice, we firstly tested the potential side effects at different concentrations administrated for at least one week. There were no obvious effects on mental state, weight and diet of the mice. Then, the anti-tumor effects of liothyronine were tested on the MC38 tumor model. We firstly selected four concentrations of liothyronine (0.5, 1.5, 5, 15 mg/kg), and 0.5 mg/kg liothyronine showed almost no anti-tumor effects, and the anti-tumor effects of 5 and 15 mg/kg liothyronine is equivalent. Then the two concentrations of 1.5 and 5 mg/kg were selected for further research (Fig. S4a). Meanwhile, anti-TIGIT antibody was also used for the tumor model, and liothyronine exhibited similar tumor inhibition with the antibodies (Fig. S4b). Subsequently, to explore the mechanism of tumor inhibition by liothyronine, MC38 tumor bearing mice were injected with normal saline, low dose (1.5 mg/kg) or high dose (5 mg/kg) of liothyronine. Both low dose and high dose of liothyronine could significantly inhibit MC38 tumor growth as above (Fig. 6a). Further, liothyronine could significantly augment the frequency of tumor infiltrating CD8^+^ T cells, and significantly enhanced the secretion of IFN-γ by tumor infiltrating CD8^+^ T cells treated with high dose of liothyronine (Fig. 6b, c). The systemic immune response was also explored, and low dose of liothyronine could significantly elicit immune responses by increasing the frequency of CD8^+^ IFN-γ^+^ T cells in the tumor draining lymph node but not in the spleen, while high dose of liothyronine could increase the frequency in both tissues (Fig. 6d, e). Thus, liothyronine could suppress tumor growth and stimulate CD8^+^ T cell response in tumor bearing mice. Since that the TIGIT/PVR immune checkpoint axis plays a major role in the functional regulation of T cells and NK cells, we also explored the role of CD4^+^, CD8^+^ T and NK cells in the tumor inhibition mediated by liothyronine therapy. T cells and NK cells depletion tumor models were established, and the depletion efficacy was verified by flow cytometry (Fig. S5). With injection of rat IgG or mouse IgG controls, liothyronine could also inhibit the MC38 tumor growth same as in Fig. 6a (Fig. 6f, g), while with the CD4^+^, CD8^+^ T and NK cells depleted by indicated depletion antibodies, the anti-tumor effects of liothyronine almost disappeared. The results demonstrated that the therapeutic effects of liothyronine depends on the existence of CD4^+^, CD8^+^ T and NK cells.

## Discussion

Although cancer immunotherapy based on PD-1/PD-L1 blockade has gained sustain progress, the low response rate and adaptive resistance make a deep investigation of other immune checkpoints urgently needed. The novel immune checkpoint TIGIT expressed on NK cells, activated CD8^+^ T cells, effector CD8^+^ T cells and regulatory T cells (Tregs), and suppressed the anti-tumor immune response by interacting with PVR [19, 20, 34]. Our previous study revealed the expression of TIGIT on colorectal tumor cells and its pivotal role in colorectal cancers [6]. Here, we found that PVR also overexpressed in many tumors inkling colorectal cancers, and the expression level increased during the tumor development. An increasing number of studies revealed that PVR was more than just a virus receptor, and it also played a critical role in a multitude of biological processes [35]. It is of great clinical significance to investigate the mechanism of TIGIT/PVR and develop inhibitors to block TIGIT/PVR.

Small molecules have entered the arena of cancer immunotherapy either by reducing immune suppression in the tumor microenvironment or enhancing activation of cytotoxic T lymphocyte responses. They have advantages over antibodies including greater penetration, oral bioavailability and fine control of bioavailability avoiding severe immune associated adverse events associated with antibodies [26]. Besides, small molecules could be easily modified or designed to combine with other cancer therapies to increase their efficacy. Small molecules dual targeting PD-L1 and VISTA (CA-170) or TIM-3 (CA-372) has been developed for cancer immunotherapy. Epacadostat, a small molecule inhibitor targeting IDO (indoleamine 2, 3-dioxygenase) has also been developed and engineered into nanoparticles for combination with photothermal therapy [36]. However, the small molecules targeting TIGIT/PVR lags far behind.

As reported, TIGIT/PVR interacts through the “key” residue of Y113 in TIGIT and F128 in PVR with the corresponding “lock” formed by AX_6_G motif, and possesses the traditional lock-and-key interface. The interface occupies most of the typical Ig β-sandwich fold formed by the A′GFCC′C′′ β-sheets [16]. Although it is quite difficult to design inhibitors that block the interaction between proteins, especially for smooth protein structures consisting of multiple β-sheets, we found a pocket suitable for small molecule occupancy on PVR. More importantly, the residues forming the pocket partially overlap with the key residues in the TIGIT/PVR binding area. Through a series of in vitro assays, liothyronine which could bind to PVR and efficiently block TIGIT/PVR interaction was obtained. We further analyzed the binding model of liothyronine and PVR, liothyronine interacts with the residues which forms the pocket with a stable structure, and occupies the TIGIT/PVR binding area. The binding model may pave the road for screening other compounds and for the optimization based on liothyronine.

SMC 14, liothyronine is a synthetic form of triiodothyronine (T3) used in the treatment of hypothyroidism, nontoxic goiter, cretinism, and myxedema. Thyroid hormones play critical role in regulating the essential biological processes including proliferation, differentiation, apoptosis, and metabolism. Although large quantities of researches have supported a relationship between thyroid hormones and the pathophysiology of various cancer types, the current studies about the relationship of T3 and colorectal cancers are complicated and uncertain [37]. Recently, a research group investigated the immune-related adverse events in non-small cell lung cancer patients treated with nivolumab (anti-PD-1 antibody), and reported that patients with low free tetraiodothyronine (fT4) in serum had significantly longer PFS and median overall survival than those without low fT4 [38]. As is well known that T4 could be catalyzed and transformed to T3, maybe liothyronine could block the TIGIT/PVR interaction and help to enhance the efficacy of ani-PD-1. It has been reported that aspirin could inhibit tumor cell growth by reducing the expression level of TIGIT, and that emodin could elicit the anti-tumor effects through downregulating the expression of PVR [39, 40]. Quite different from that, liothyronine could exert anti-tumor effects by TIGIT/PVR blockade. Liothyronine could block both human and mouse TIGIT/PVR interaction, although the blocking efficacy of mouse TIGIT/PVR is not so obvious. The differences of the efficacy may be attributed to that liothyronine has better binding affinity to human PVR than mouse PVR. Thus, through the significantly tumor inhibition on tumor model, we could infer that liothyronine may have better results in human. Different from the traditional chemotherapy drugs, liothyronine has no influence on the proliferation of tumor cells. TIGIT/PVR blockade by liothyronine could reverse the inhibition of IL-2 secretion of Jurkat cells and enhance the function of T cells in the activated PBMCs. The anti-tumor effects of liothyronine in the MC38 tumor model also relies on its function on immune cells. Liothyronine could increase the quantities and functional quality of tumor infiltrating CD8^+^ T cells, and exert systemic immune response. Besides, the immune cell depletion models confirmed that the anti-tumor effects of liothyronine depends on the existence of T cells and NK cells. These results suggested that liothyronine had the similar characteristics as other immune checkpoint blockers.

## Conclusions

Here, we confirmed the overexpression of PVR on a broad spectrum of cancers, especially in colorectal cancers. We also developed the first small molecule, liothyronine, targeting PVR by docking-based virtual screening. Liothyronine could bind to PVR and block the interaction of TIGIT/PVR, as well as enhance the function of T cells in vitro. It also exerted anti-tumor effects by augmenting the tumor infiltrating quantity and function of CD8^+^ T cells. Our research provides a promising candidate for cancer immunotherapy based on TIGIT/PVR blockade.

## Supplementary information

**Additional file 1: Figure S1.** The binding affinity of SMC 14 to human and mouse PVR by MST assay. **Figure S2.** The binding model of liothyronine with PVR. **Figure S3.** The blocking efficacy of PD-1/PD-L1 and CD47/Sirpα interaction by liothyronine. **Figure S4.** The anti-tumor effects of liothyronine and anti-TIGIT antibody on MC38 tumor model. **Figure S5.** The depletion efficacy of CD4^+^ T, CD8^+^ T and NK cells in MC38 tumor model. **Table S1.** The binding pocket for small molecules on PVR. **Table S2.** Candidate compounds through virtual screening.

## Data Availability

The datasets generated during and/or analyzed during the current study are available from the corresponding author on reasonable request.

## Abbreviations

TIGIT: T cell immunoglobulin and immunoreceptor tyrosine-based inhibitory motif domain
PVR: Poliovirus receptor
GEO: Gene Expression Omnibus
TCGA: The Cancer Genome Atlas
MOE: Molecular operating environment
BLI: Biolayer interferometry
PBMCs: Peripheral blood mononuclear cells
MTT: 3-(4, 5-dimethyl-2-thiazolyl)-2,5-diphenyl-2-H-tetrazolium bromide
T3: Triiodothyronine
T4: Tetraiodothyronine
