# Supplementary material for: Repositioning liothyronine for cancer immunotherapy by blocking the interaction of immune checkpoint TIGIT/PVR
Source: Cell Commun Signal. 2020 Sep 7;18:142. doi: 10.1186/s12964-020-00638-2 (PMC7487564; doi:10.1186/s12964-020-00638-2)
Supplement: Supplementary file 2 — Additional file 1: Figure S1. The binding affinity of SMC 14 to human and mouse PVR by MST assay. Figure S2. The binding model of liothyronine with PVR. Figure S3. The blocking efficacy of PD-1/PD-L1 and CD47/Sirpα interaction by liothyronine. Figure S4. The anti-tumor effects of liothyronine and anti-TIGIT antibody on MC38 tumor model. Figure S5. The depletion efficacy of CD4+ T, CD8+ T and NK cells in MC38 tumor model. Table S1. The binding pocket for small molecules on PVR. Table S2. Candidate compounds through virtual screening. [file 12964_2020_638_MOESM2_ESM.docx]

**Supplemental Materials.**

There are five supplemental figures and two supplemental tables.

**Supplemental figures and legends**

**Figure S1. The binding affinity of SMC 14 to human and mouse PVR by MST assay.** Human and mouse PVR protein fused with His tag were labeled with Red-NHS647 dye. SMC 14 was 2-fold serially diluted from 100 μM and 16 samples were subsequently tested. Equal volume of dye-labeled protein was incubated with the diluted SMC 14 and loaded onto standard capillaries for detection. The K_D_ values were calculated with analysis software (MO.Affinity Analysis v2.2.4). Data are representative of at least three independent experiments.

**Figure S2. The binding model of liothyronine with PVR.** Liothyronine occupies the binding pocket (red surface) and partial of the TIGIT/PVR binding area. The 2D and 3D interaction diagram were shown.

**Figure S3. The blocking efficacy of PD-1/PD-L1 and CD47/Sirp**α **interaction by liothyronine.** (A) The representative FACS histogram of blocking assay of liothyronine (100 μM) with CHOK1-hPD-1 cells and PD-L1-Fc protein (blue histogram). (B) The representative FACS histogram of blocking assay of liothyronine (100 μM) with CHOK1-hCD47 cells and Sirpα-Fc protein (blue histogram).

**Figure S4.** **The anti-tumor effects of liothyronine** **and anti-TIGIT antibody on MC38 tumor model.** (a) Tumor growth curve of MC38 tumor-bearing mice treated with normal saline, 0.5, 1.5, 5 or 15 mg/kg of liothyronine by intraperitoneal injection every other day. (*n*=5-6, **P*< 0.05, ***P*<0.01). (b) Tumor growth curve of MC38 tumor-bearing mice treated with 200 μg anti-TIGIT or mouse IgG control by intraperitoneal injection every three days. (*n*=5, ***P*<0.01).

**Figure S5. The depletion efficacy CD4^+^ T, CD8^+^ T and NK cells in MC38 tumor model.** Blood samples of the mice treated with anti-CD4, anti-CD8, anti-NK1.1 or matched IgG controls for six days were used to analyze the depletion efficacy by flow cytometry. Data are representative of at least three independent experiments.

Figure S1.


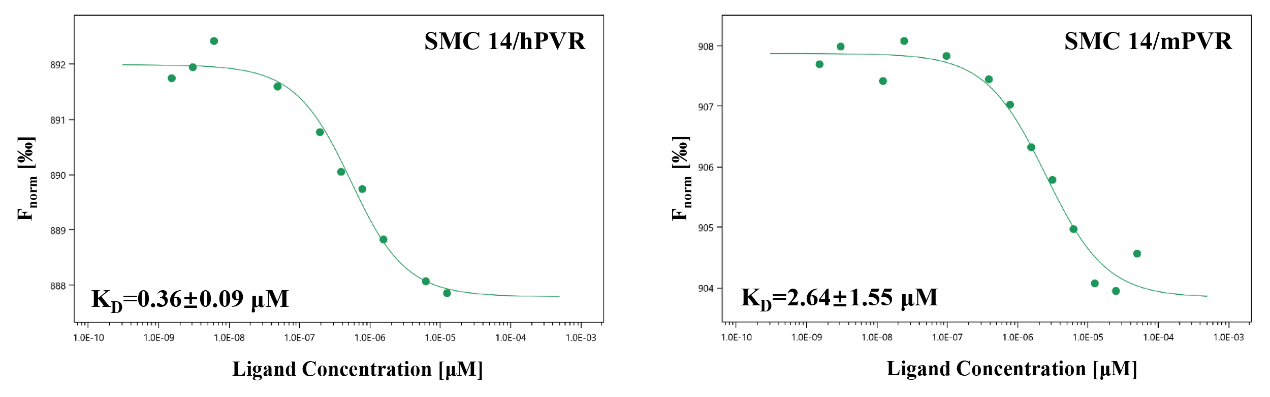


Figure S2.


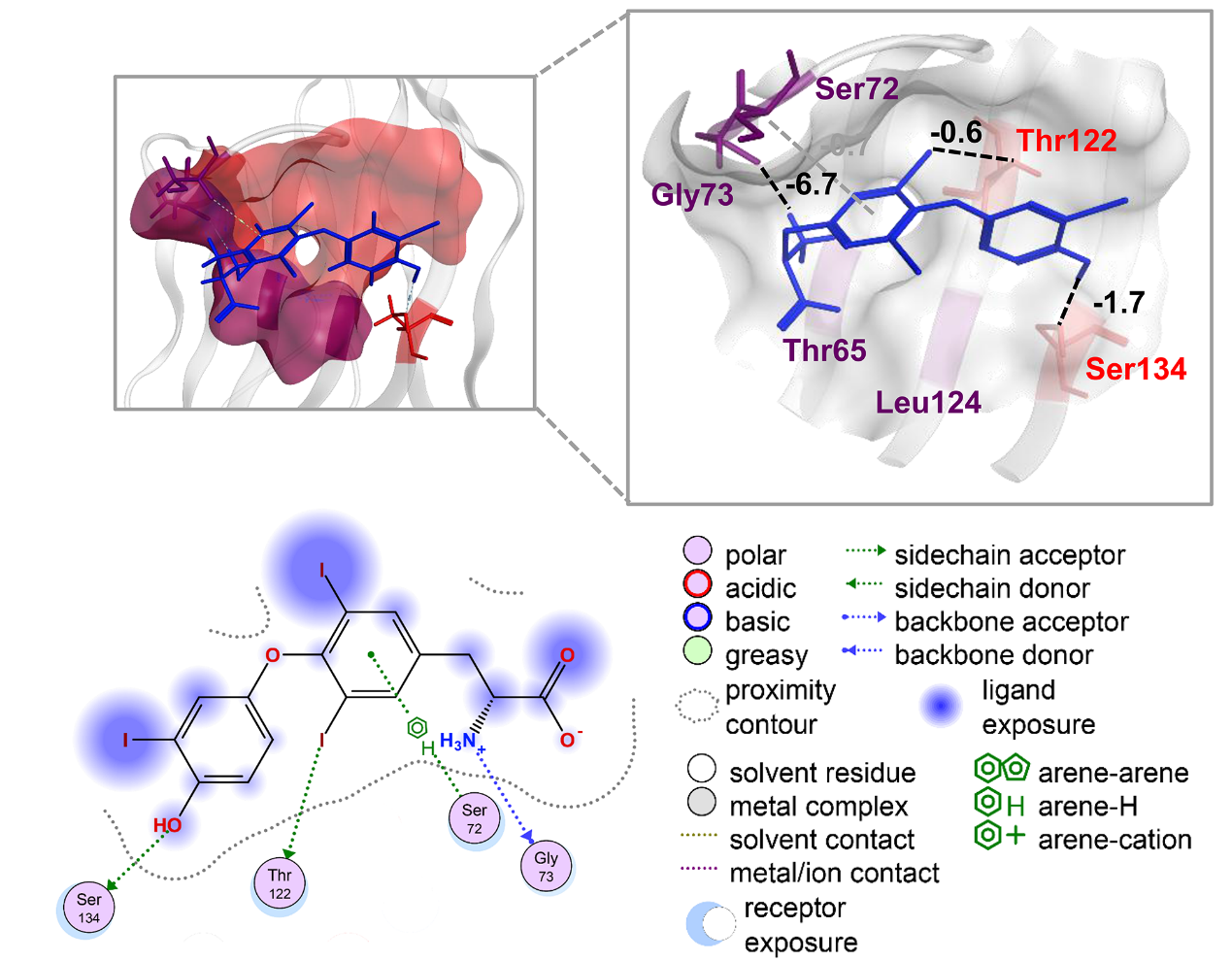


Figure S3.


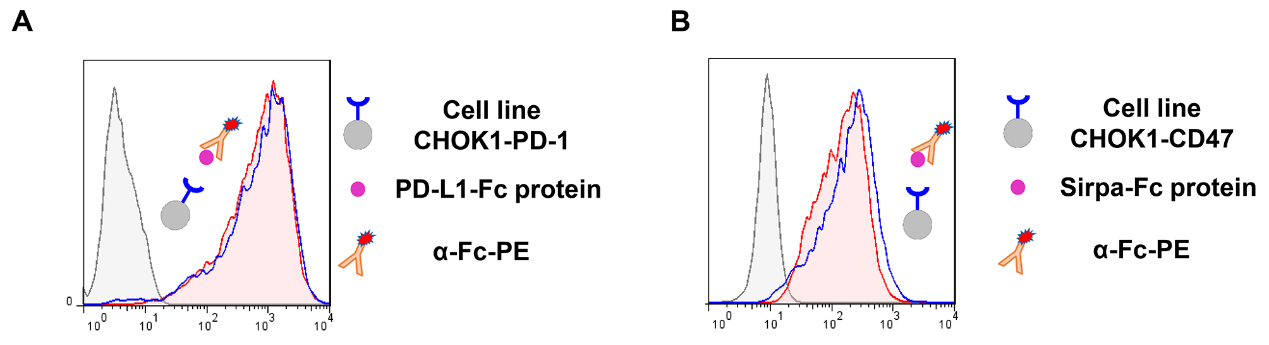


Figure S4.


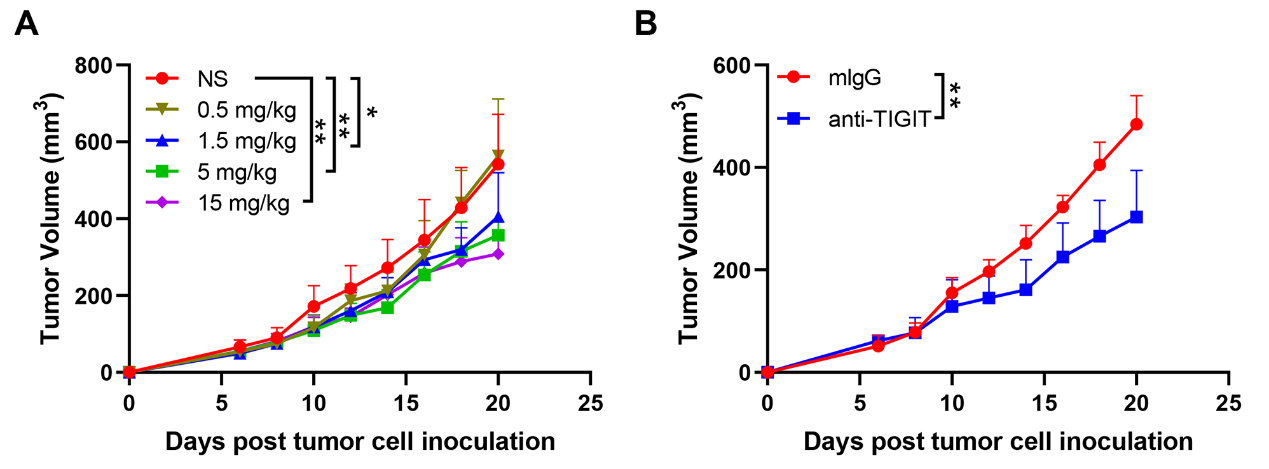


Figure S5.


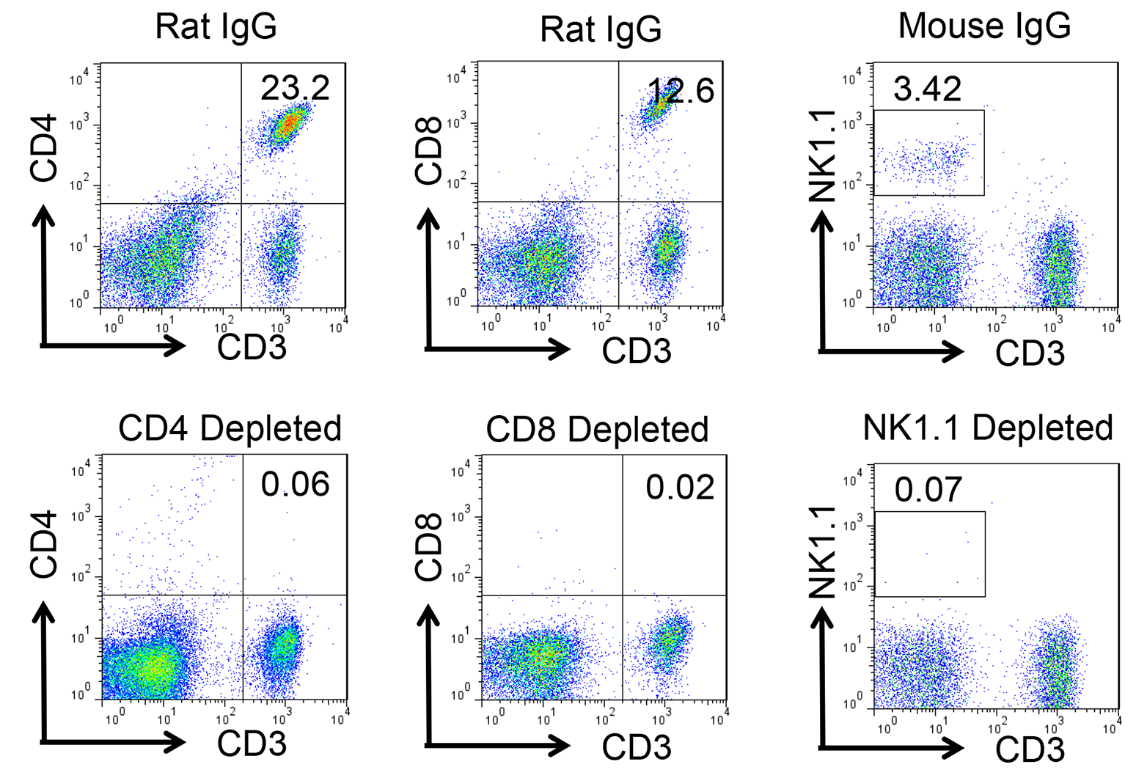


Table S1. The binding pocket for small molecules on PVR.

| **Number** | **Size** | **PLB** | **Hyd** | **Side** | **Residues** |
| --- | --- | --- | --- | --- | --- |
| 1 | 51 | 1.63 | 30 | 76 | A67,R68,E71,S72,G73,S74,M75,S87,E88,S89,R91,M110,E116,D117,Y121 |
| 2 | 18 | 0.15 | 8 | 30 | T65,A67,R68,H69,G70,E71,G73,T122,L124,D136 |
| 3 | 18 | 0.11 | 10 | 26 | T50,L51,Q52,V53,P54,N55,M56,E57,V58,T59,E102,R104 |
| 4 | 16 | -0.44 | 12 | 16 | H60,V61,Q80,R98,L99,G10,A101,E102,L103 |
| 5 | 7 | -1.45 | 10 | 12 | Y86,E88,S89,K90 |

The pocket was identified by using the Site Finder module of MOE. The Size column indicates the number of alpha spheres comprising the site. The PLB column indicates the propensity for ligand binding score for the contact residues in the receptor. The Hyd column indicates the number of hydrophobic contact atoms in the receptor. The Side column indicates the number of sidechain contact atoms in the receptor. The Residues column indicates the residues that make up the calculated site in the format chain. residue-name.

Table S2. Candidate compounds through virtual screening.

| **SMC** | **Name** | **Structure** | **Weight** | **S-score** | **Interactions** |
| --- | --- | --- | --- | --- | --- |
| 1 | Harmine | 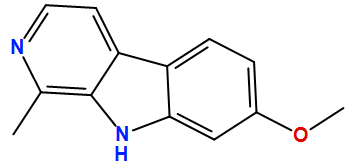 | 212.3 | -4.23 | E71,-1.1 |
| 2 | Indigo | 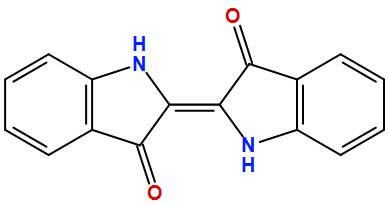 | 262.3 | -4.25 | G70,-3.3 |
| 3 | Vitamin B1 | 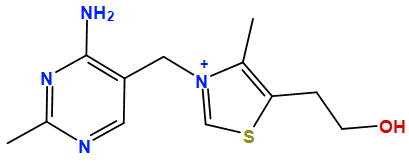 | 265.4 | -4.81 | G70,-2.1; E71,-2.7; G73,-1.6 |
| 4 | Capsaicin | 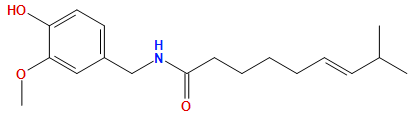 | 305.4 | -4.51 | S134,-0.8 |
| 5 | Glutathione | 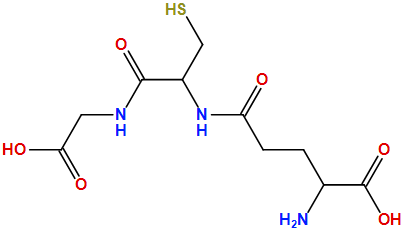 | 307.3 | -5.30 | G73,-1.2 |
| 6 | Curcumin | 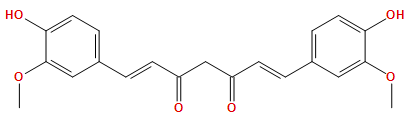 | 368.4 | -5.47 | G70,-0.9 |
| 7 | Pyrithioxin | 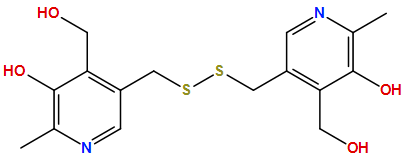 | 368.5 | -5.54 | E71,-0.8 |
| 8 | Mevastatin | 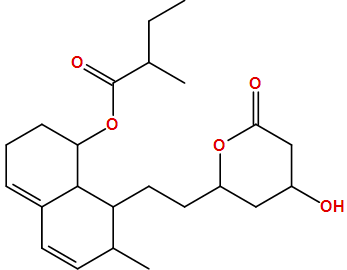 | 390.5 | -5.61 | S72,-2.3 |
| 9 | Riboflavin phosphate sodium | 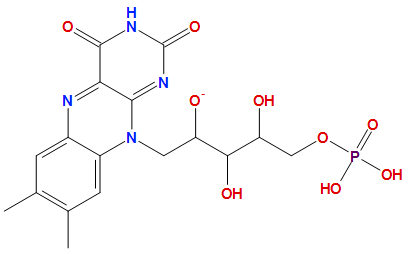 | 455.3 | -5.49 | G70,-1.7; D136,-9.3 |
| 10 | Paeoniflorin | 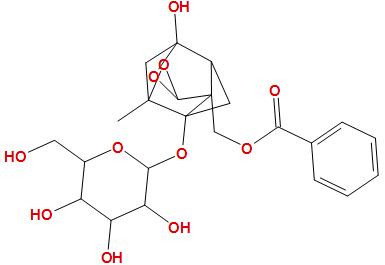 | 480.5 | -5.03 | D136,-2.1 |
| 11 | Taurocholic acid sodium salt hydrate | 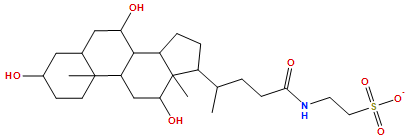 | 514.7 | -5.66 | S134,-1.2 |
| 12 | Forsythin | 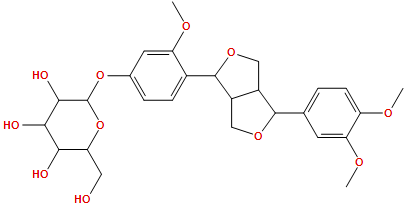 | 534.6 | -6.85 | Q63,-0.6; G70,-2.8 |
| 13 | Bilirubin | 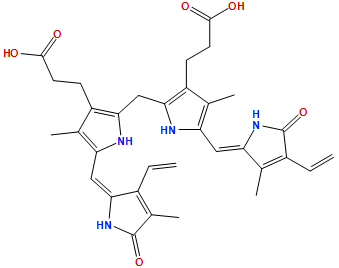 | 584.7 | -6.36 | H79,-2.1; S132,-1.7 |
| 14 | Liothyronine | 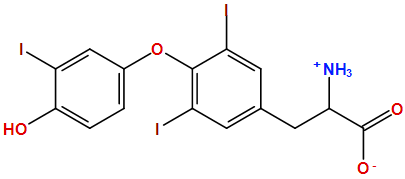 | 651.0 | -4.72 | G73,-6.7; T122,-0.6;S134,-1.7 |
| 15 | Puerarin | 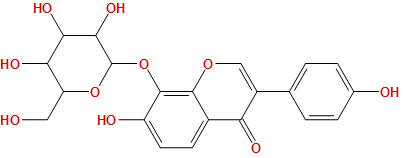 | 432.4 | -5.88 | Q63,-1.1 |
| 16 | Griseofulvin | 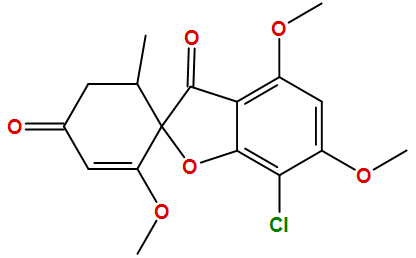 | 352.8 | -4.96 | Q63,-2.3 |
| 17 | Cordycepin | 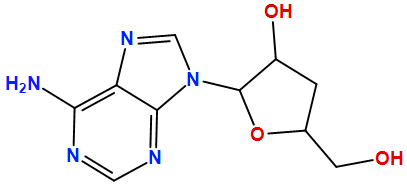 | 251.2 | -4.93 | H69,-2.2; G70,-3.6 |
| 18 | Dihydromyricetin | 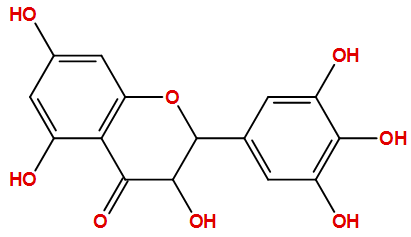 | 320.3 | -4.63 | G73,-1.3 |
| 19 | Olaquindox | 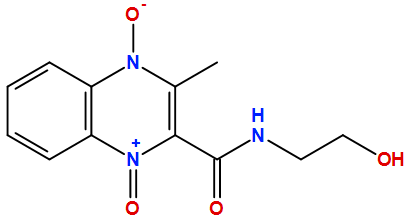 | 263.3 | -4.76 | G70,-4.8; G73,-1.3 |
| 20 | S-allyl-L-cysteine | 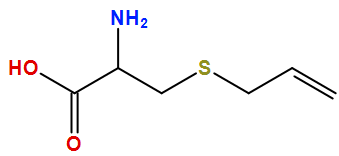 | 161.2 | -4.70 | E71,-1.7; T122,-1.9 |
| 21 | Salvianolic-Acid-C | 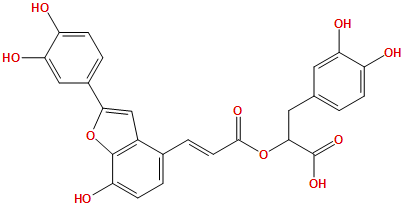 | 492.4 | -4.70 | T65,-0.8; S72,-2 |
| 22 | Epicatechin gallate | 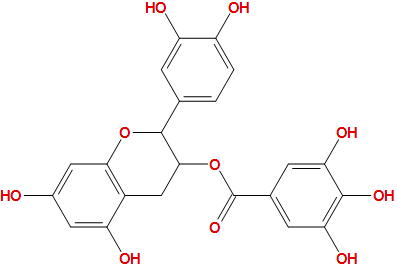 | 442.4 | -4.67 | E71,-0.9 |
